# Neonatal Sepsis Diagnosis Decision-Making Based on Artificial Neural Networks

**DOI:** 10.3389/fped.2020.00525

**Published:** 2020-09-11

**Authors:** Addy Cecilia Helguera-Repetto, María Dolores Soto-Ramírez, Oscar Villavicencio-Carrisoza, Samantha Yong-Mendoza, Angélica Yong-Mendoza, Moisés León-Juárez, Jorge A. González-y-Merchand, Verónica Zaga-Clavellina, Claudine Irles

**Affiliations:** ^1^Department of Immunobiochemistry, Instituto Nacional de Perinatología, Mexico City, Mexico; ^2^Department of Microbiology, Escuela Nacional de Ciencias Biológicas, Instituto Politécnico Nacional, Mexico City, Mexico; ^3^Department of Physiology and Cellular Development, Instituto Nacional de Perinatología, Mexico City, Mexico

**Keywords:** newborn, neural network, sepsis, machine learning, prematurity

## Abstract

Neonatal sepsis remains difficult to diagnose due to its non-specific signs and symptoms. Traditional scoring systems help to discriminate between septic or not patients, but they do not consider every single patient particularity. Thus, the purpose of this study was to develop an early- and late-onset neonatal sepsis diagnosis model, based on clinical maternal and neonatal data from electronic records, at the time of clinical suspicion. A predictive model was obtained by training and validating an artificial Neural Networks (ANN) algorithm with a balanced dataset consisting of preterm and term non-septic or septic neonates (early- and late-onset), with negative and positive culture results, respectively, using 25 maternal and neonatal features. The outcome of the model was sepsis or not. The performance measures of the model, evaluated with an independent dataset, outperformed physician's diagnosis using the same features based on traditional scoring systems, with a 93.3% sensitivity, an 80.0% specificity, a 94.4% AUROC, and a regression coefficient of 0.974 between actual and simulated results. The model also performed well-relative to the state-of-the-art methods using similar maternal/neonatal variables. The top 10 factors estimating sepsis were maternal age, cervicovaginitis and neonatal: fever, apneas, platelet counts, gender, bradypnea, band cells, catheter use, and birth weight.

## Introduction

Neonatal sepsis is a syndrome featured by non-specific signs and symptoms of systemic infection accompanied by bacteremia in the first 28 days of extra uterine life (1). This condition is a public health problem that still contributes to mortality and morbidity in neonatal intensive care units (NICUs) in high (2–4), as well as low- and middle-income countries (5–7). Thus, in order to diminish neonatal deaths, early pharmacological therapy must be initiated; unfortunately, antibiotics are not specific, getting an accurate diagnosis even for experienced clinicians is difficult due to the non-specific signs and symptoms, and may take several days to get the culture test results (8, 9). Moreover, sepsis consensus definitions, like the new adult Sepsis-3 definition (10), are not designed for either preterm or term neonates (4). Therefore, clinicians must start empirical antibiotic therapies based on a combination of maternal and neonatal risk factors, as well as clinical signs and symptoms, that do not take into account the particularities between every single patient. Clinical algorithms include maternal (urinary tract and sexually transmitted infections, premature rupture of membranes, intrapartum fever, chorioamnionitis, and/or malodorous discharge) and neonatal risk factors (prematurity and low birthweight), as well as neonatal signs and symptoms including (temperature >37.7 or >35.5°C, bradycardia, respiratory rate > 20/min or hypotension, tachycardia, apnea, hemodynamic instability, and abnormal laboratory data: white blood cell counts > 12,000/mm3 or < 4,000/mm3) (1, 11). However, neonatal sepsis management also results in an antibiotic intervention of uninfected infants that might lead to adverse outcomes (12–14); thus the development of new neonatal sepsis diagnostic tools that consider individual combinations of clinical variables is warranted (15). Molecular techniques have been proposed (8); nevertheless, all of them require invasive neonatal blood drawn, special equipment, trained technicians, which are expensive and unavailable in many low- and middle-income countries. Given the challenge to diagnose sepsis, over the last few years, statistical and computational tools have emerged as cost-effective strategies to establish a faster diagnosis and decision-based clinical guidelines (16).

Artificial intelligence, particularly Artificial Neural Networks (ANN) are robust methodologies for the forecasting of diagnosis/prognosis with high predictive accuracy, and nowadays are used to support medical decisions in NICUs (17, 18). Different computational models have been developed to predict adult, pediatric and neonatal sepsis (19–23). Neonatal sepsis diagnosis may be considered a customizable condition, mostly in preterm patients with unique obstetric situations such as maternal and fetal/neonatal morbidities. Therefore, in support of decision-making for clinicians the day of sepsis suspicion, and as an additional non-invasive test, the aim of this study was to develop a forecasting model of early-onset (<48–72 h) and late-onset (>72 h) sepsis (EOS and LOS) diagnosis at the time of clinical suspicion in the NICU. The model was based on maternal-neonatal signs and symptoms from clinical information, including known risk factors, anthropometric, laboratory data, as well as maternal and fetal/neonatal morbidities from electronic records.

## Materials and Methods

### Ethical Approval and Study Design

This observational retrospective study was approved by the Institutional Review Boards of the Instituto Nacional de Perinatología Isidro Espinosa de los Reyes (INPerIER), Research and Ethics Committees (#2017-2-65 to CI and #212250-3210-11007-04-14 to ACHR) in accordance with the Helsinki Declaration. Data were collected over a period of 18 months from medical records of 236 neonates hospitalized in the Neonatal Intensive Care Unit (NICU) of the INPerIER, a tertiary care hospital, in which patient names and identifiers were eliminated in order to have an anonymized dataset. Informed consent was not required.

The main purpose of this work was to obtain a model of neonatal sepsis diagnosis (including EOS and LOS), at the time of clinical suspicion in the NICU by an artificial neural network approach, for which we collected medical records from mothers and neonates. The secondary objective of this study was to classify maternal and neonatal factors in accordance to their importance for sepsis diagnosis from the developed model. Preterm and term infants were classified as either: (1) Not-septic, with negative culture results (neonates with suspicion of sepsis but finally diagnosed by clinicians as not septic; treated or not with antibiotics) or (2) Sepsis, with confirmed positive culture results (treated or not with antibiotics), both groups with clinical signs and symptoms of sepsis. Exclusion criteria were chromosomal abnormalities and genetic syndromes. Population characteristics are depicted in Table 1.

**Table 1:** Clinical characteristics of the study population for the selected clinical features.

	**Without Sepsis (*n* = 132)** **(mean, range)**	**Sepsis (*n* = 106)** **(mean, range)**
Maternal age (years)	29 (15–49)	27 (14–47)
Cervicovaginitis (%)	No 68 (51.5%) Yes 64 (48.5%)	No 36 (34.95%) Yes 67 (65.05%)
Urinary tract infections (%)	No 54 (40.9%) Yes 78 (59.1%)	No 35 (33.98%) Yes 68 (66.02%)
Premature rupture of membranes (%)	No 89 (67.4%) Yes 43 (32.6%)	No 71 (68.93%) Yes 32 (31.07%)
Chorioamnionitis (%)	No 121 (91.7%) Yes 11 (8.3%)	No 91 (88.35%) Yes 12 (11.65%)
Gender (%)	Male 68 (51.5%) Female 64 (48.5%)	Male 53 (51.46%) Female 50 (48.54%)
Gestational age (weeks)	33.49 (27.5–40.4)	30.6 (25–40.4)
Weight (grams)	1,842 (755–4,935)	1,291 (520–3,605)
Fever (%)	No 115 (87.1%) Yes 17 (12.9%)	No 72 (69.90%) Yes 31 (30.10%)
Hypothermia (%)	No 130 (98.5%) Yes 2 (1.5%)	No 88 (85.44%) Yes 15 (14.56)
Tachycardia (%)	No 124 (93.9%) Yes 8 (6.1%)	No 69 (69.99%) Yes 34 (33.01%)
Bradycardia (%)	No 130 (98.5%) Yes 2 (1.5%)	No 101 (98.06%) Yes 2 (1.94%)
Tachypnea (%)	No 121 (91.7%) Yes 11 (8.3%)	No 74 (71.84%) Yes 29 (28.16%)
Bradypnea (%)	No 115 (87.1%) Yes 17 (12.9%)	No 103 (100.00%) Yes 0 (0%)
Apnea (%)	No 97 (73.5%) Yes 35 (26.5%)	No 72 (69.90%) Yes 31 (30.10%)
Leukocytes (cells/mm^3^)	12,631 (3,900–110,000)	12,254 (2,300–33,200)
Neutrophils (cells/mm^3^)	5,844 (876–39,058)	7,239 (646–24,336)
Band cells (cells/mm^3^)	248 (0–3,972)	713 (40–3,240)
Band cells (%)	2 (0–12)	5 (0.02–20)
Relation band/neutrophils	0.044 (0–0.250)	0.12 (0.015–0.8)
Platelets (cells/mm^3^)	275,013 (17,700–799,000)	159,716 (242,000–593,000)
Catheter (%)	No 36 (27.3%) Yes 96 (72.7%)	No 22 (21.36%) Yes 81 (78.64%)
Mechanical ventilation (%)	No 39 (29.5%) No invasive 79 (59.9%) Invasive 14 (10.6%)	No 27 (26.21%) No invasive 59 (57.28%) Invasive 17 (16.51%)
Received initial pharmacological treatment (%)	No 65 (49.2%) Yes 67 (50.8%)	No 1 (0.97%) Yes 102 (99.03%)

### Dataset

We carefully composed a balanced dataset with a similar number of infants in the non-sepsis and sepsis groups from medical records of the NICU, to train and evaluate the model. Medical records with incomplete data as well as neonates with suspicion of sepsis with negative culture and diagnosis of clinical sepsis were excluded. The dataset was created by merging records from the clinical, anthropometric, microbiology, antibiotics, laboratory, and NICU documentation. We selected clinical signs/symptoms and risk factors for neonatal sepsis based on the same criteria as traditional scoring systems used by clinicians, and well-known risk factors through literature revision. In addition, maternal and fetal morbidity were also taken into account as features for the model.

### Learning, Testing, and Validation of the Model

**Figure 1:**
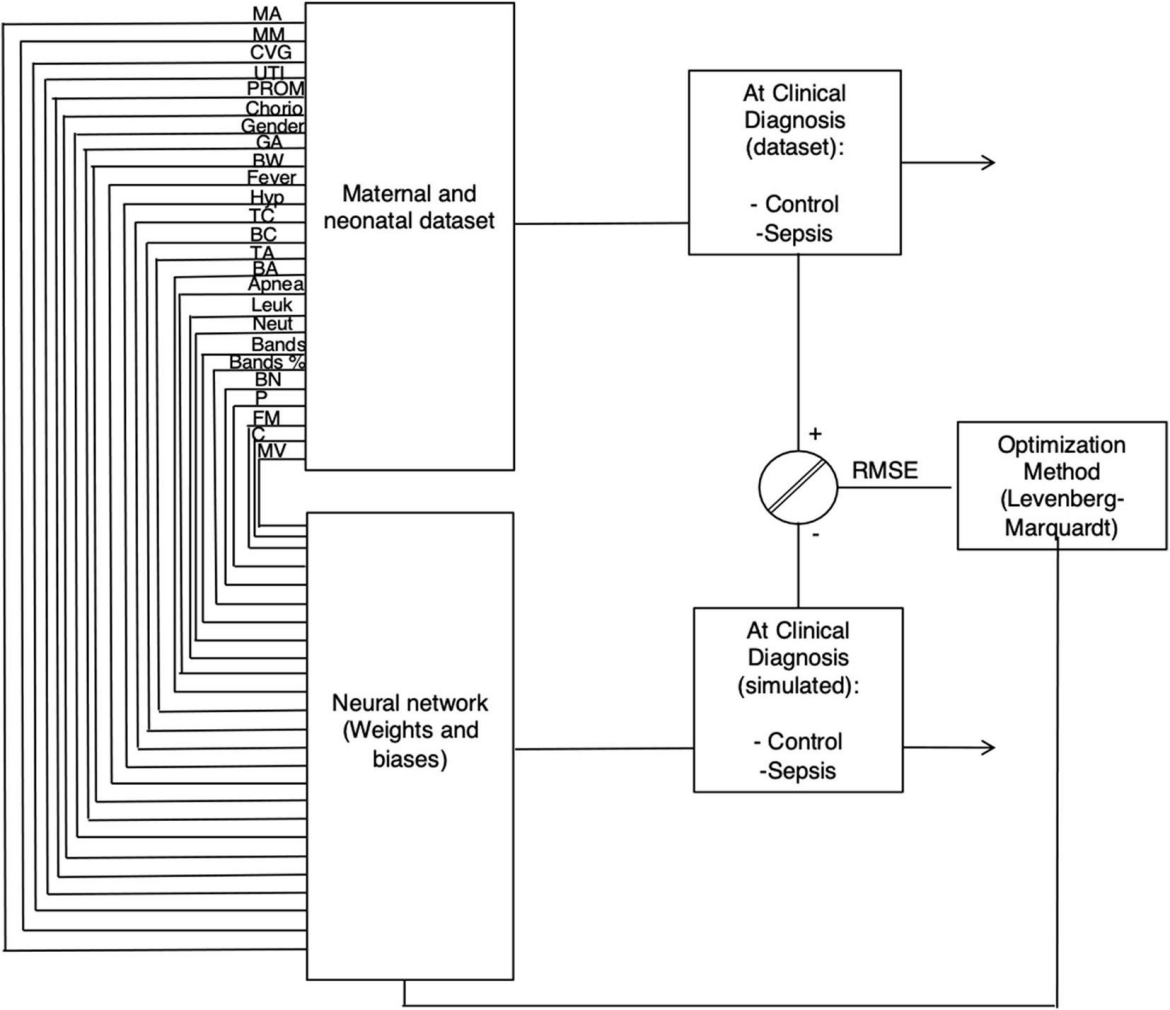
The network architecture for the clinical sepsis prediction model. Twenty-five maternal and neonatal variables at clinical diagnosis (Maternal age, maternal morbidity, Cervicovaginitis, Urinary tract infections, Premature rupture of membranes, Chorioamnionitis, Gender, Gestational age, neonatal weight, Fever, Hypothermia, Tachycardia, Bradycardia, Tachypnea, Bradypnea, Apneas, number of leukocytes and neutrophils, Band cells number and percentage, Platelets number, Platelet, fetal morbidity, Catheter, and Mechanical ventilation) were used to for the learning procedure with the Levenberg-Marquardt optimization algorithm and sepsis estimation as the output of the model.

A neural network consists of an input layer with maternal and neonatal features (neurons) connected through coefficients (Weights and biases, W and b, respectively), to the hidden and output layers (outcome of the model: non-sepsis or sepsis) to form a network. Figure 1 shows the structure of the ANN model and equations are found in Supplementary Material.

Maternal variables were: age (MA), morbidity (MM) (Table S1 in Supplementary Material), presence or absence of Cervicovaginitis (CVG), urinary tract infections (UTI), premature rupture of membranes (PROM), and chorioamnionitis (Chorio).

Cervicovaginitis was diagnosed by microbiology cultures for the most prevalent pathogens, including *Ureaplasma, Mycoplasma, Chlamydia, Candida* sp, *Trichomonas*, and others that causes vaginosis such as *Gardnerella*. Presence of GBS (group B Streptococci) was also analyzed.

Chorioamnionitis was diagnosed using histological examinations and was described as the presence of inflammatory lesions of the placenta with infiltration of neutrophils at different sites of the organ. This definition includes acute chorioamnionitis, funisitis, and chorionic vasculitis representing the immune response of fetal and maternal tissues (24).

Neonatal factors were: gestational age (GA), gender, birth weight (BW), presence or absence of clinical symptoms associated with sepsis diagnosis such as fever, hypothermia (Hypo), tachycardia (TC), bradycardia (BC), tachypnea (TA), bradypnea (BA), apneas; laboratory findings: number of leukocytes (Leuk), neutrophils (Neut), band cells (Bands) and bands percentage (% Band), ratio of band neutrophils to segmented neutrophils or immature/total neutrophil ratio (BN), platelets (P) as well as fetal/neonatal morbidity (FM) (Table S2 in Supplementary Material), presence or absence of catheter (C) and mechanical ventilation (MV: without, invasive-orotracheal intubation- or not invasive). There were no missing values. Only input variables were normalized between 0.1 and 0.9, as previously described (25).

Hypothermia was defined as a temperature <35.5°C; bradycardia as a respiratory rate > 20/min; tachycardia as a heart rate >160; tachypnea as a respiratory rate >60 per min; bradypnea as a respiratory rate of fewer than 30 breaths per minute; apnea as the cessation of breath for at least 20 or <20 s in presence of bradycardia.

We first randomly set aside 15% of the total database that will be used to evaluate the final model (test cohort) by measuring its ability to discriminate between those with and without sepsis, employing the Area Under the Receiver Operating Characteristics Curve (AUROC) as the metric of choice (see section Evaluation of the final ANN neonatal sepsis model by performance measures: discrimination in comparison with the physician's diagnosis). The 85% of the remaining database was split into training (learning set, 70%), validation and testing sets (15%).

In order to evaluate the accuracy of the model during training, we used a 5-fold-cross-validation, the specifics of the methodology and equations are provided as Supplementary Material. Briefly, the model was trained with a set of data (to adjust the Weights on the network), which is different to the validation set (used to tune the architecture of the model such as number of neurons and mathematical functions in the hidden layer after each training), and to the test set (which evaluates the predictive power of the finished model). Training, validation and testing of the model were done with back-propagation (BP) neural networks (26, 27) programmed with MatLab software (Natick, MS, USA) with the Neural Network toolbox (we did not use the Matlab interface Neural network getting started GUI - MATLAB nnstart).

During training and validation, three metrics were used to evaluate the performance of the model. The error (or Root Mean Square Error, RMSE), the regression coefficient (*R*^2^), and the statistical slope and intercept [section Statistical test for internal validation (slope and intercept test), (28)] between the actual (experimental) and the simulated (target) data. In order to adjust the coefficients (weights and biases), the Levenberg-Marquardt (LM) algorithm (29) was applied in order to minimize the RMSE. The LM gives accurate training results for moderate size neural networks and higher convergence speed [equation (3), Supplementary Material].

In order to find the best performance, several activation functions (linear, Log-sigmoid or hyperbolic tangent) were applied in the hidden and output layers for the learning procedure, since one transfer function may outperform the other in fitting the data. We begin training with one neuron until the RMSE was <10^−12^ and did not change, and the statistical test [slope and intercept, see Section Statistical test for internal validation (slope and intercept test)] was approved. For details see (25, 30, 31). The program was run 30,000 times with 100 iterations by each neuron.

### Statistical Test for Internal Validation (Slope and Intercept Test)

Linear regression between the experimental values (training and validation) and the ANN predicted values was performed in order to obtain the best performance of the ANN sepsis model, evaluated by the slope and intercept statistical test (28). The range of the slope and the intercept in the linear regression must be closer to 1 and zero, respectively, with a 99.8% confidence level in accordance with the Student *t*-test.

### Evaluation of the Final ANN Neonatal Sepsis Model by Performance Measures: Discrimination in Comparison With the Physician's Diagnosis

The sepsis model is a binary prediction problem: sepsis or not. Therefore, standard measures of model performance, including the Area under the Receiver Operating Characteristics curve (AUROC), accuracy, precision, sensitivity, specificity, negative, and positive predictive values (NPD and PPV, respectively), were conducted for the final model on a different database from the learning, validation, and testing sets (test cohort). These metrics have been currently used to evaluate the performance of machine learning models (32) in order to measure the predictive capacity to diagnose sepsis in a more generalized context with new data. The equations for accuracy, precision, sensitivity, and specificity are depicted as Supplementary Material.

The ROC curve was done with Graphpad Prism 5 software (San Diego, CA, USA).

### Sensitivity Analysis

In order to obtain the relative importance of each input variable for the prediction of sepsis, we performed a sensitivity analysis based on the partitioning of connection weights and the Garson algorithm (33), as previously described (25).

## Results

### Characteristics of the Study Population

Since blood culture remains the gold standard for sepsis confirmation but takes several days before results are acquired, the aim of this study was to develop an ANN model of sepsis diagnosis discriminating between newborns with clinical signs and symptoms of sepsis but with or without confirmed sepsis by culture results, in support of a medical decision the day of suspicion. We included preterm and term neonates with similar clinical signs and symptoms of early- and late-onset sepsis (EOS and LOS) but with or without sepsis (Table 1). A total of 238 neonates, including 132 non-septic and 106 sepsis confirmed (culture-positive results, 22 EOS and 84 LOS) infants were used for the balanced database after 3,508 infants with an outcome other than sepsis, and 10 neonates with incomplete medical records were excluded from the analysis. Mean gestational age for sepsis and non-septic neonates was 27.5–40.4 and 25–40.4 weeks, respectively.

The model was created by training an ANN algorithm with maternal and neonatal variables associated with sepsis, including well-known risk factors, laboratory, and signs/symptoms from traditional scoring systems, as well as the outcome of sepsis or not from the database.

### Development and Internal Validation of Neonatal Sepsis Diagnosis Model

**Figure 2:**
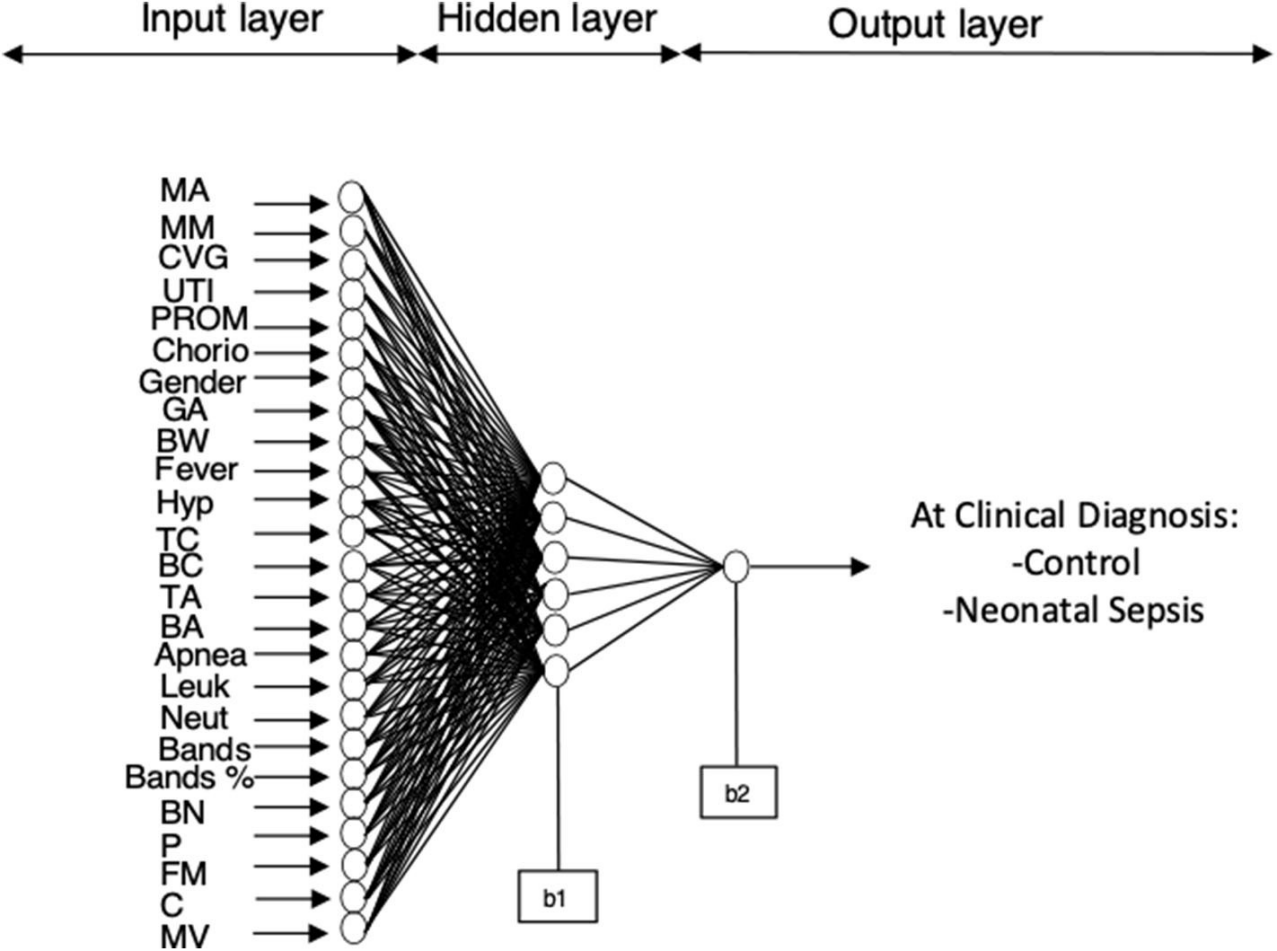
ANN model for sepsis estimation on the day of clinical diagnosis. The final architecture of the model was 25 neurons in the input layer, 6 neurons in the hidden layer and 1 neuron in the output layer (25-6-1).

The best performing model for sepsis diagnosis after training and validation consisted of a neural network with the 25 maternal and neonatal features connected through coefficients (W and b) to a hidden layer (6 neurons) and the output layer which is the outcome: sepsis or not (Figure 2). Diagnosis of sepsis is codified as one and non-sepsis as zero.

**Figure 3:**
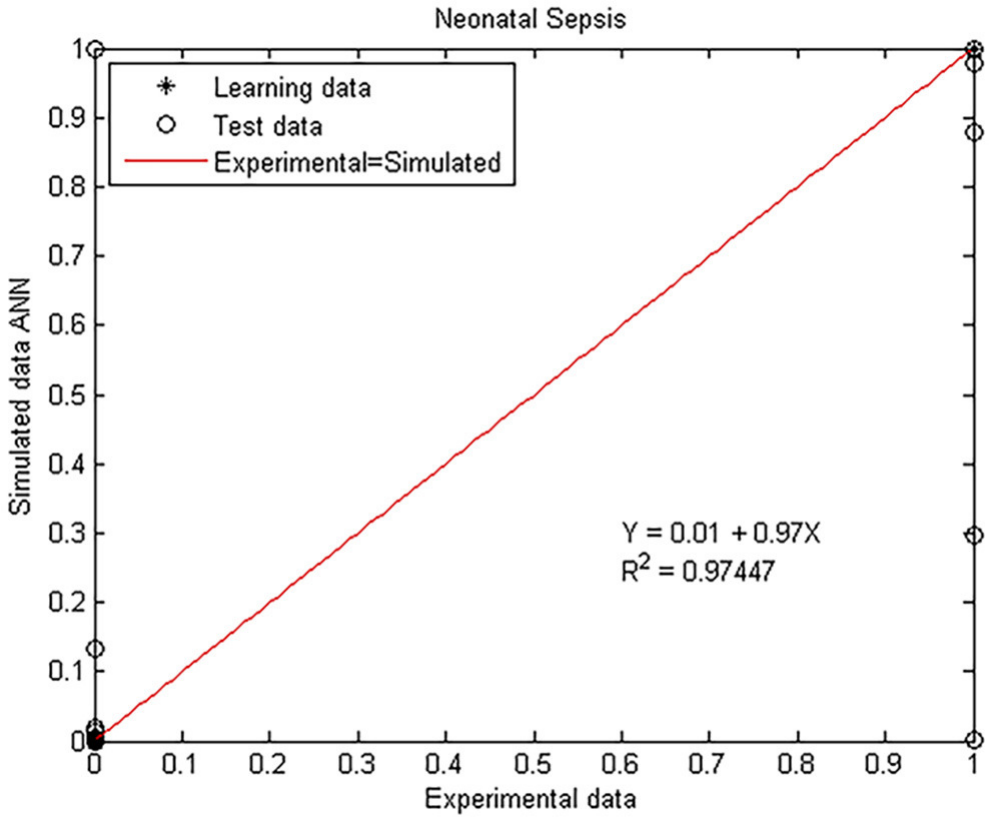
Experimental vs. ANN simulated values for sepsis estimation on the day of clinical diagnosis. Scatter plot of the model where open circles and closed diamonds show experimental and learning data, respectively, and red lines indicate the linear regression model on scatter points.

Figure 3 depicts the experimental (actual) values compared to the simulated numbers in the testing and internal validation database. Differences between real and predicted values analyzed through a linear regression model shows a regression coefficient of *R*^2^ = 0.974. The measures of the statistical test applied to the regression showed that the slope and intercept were near to 1.0 and 0, respectively (Table S3, in Supplementary Material), with a 99.8% confidence.

Based on the score from the linear regression, infants are therefore diagnosed with sepsis when the value of the prediction is >0.85 and non-sepsis below this number (Figure 3).

Sepsis diagnosis the day of clinical suspicion can be calculated based on an equation (with Weights and biases from Table S4, see Supplementary Material).

### Assessment of Prediction Ability: Model Performance Compared to the Physician's Diagnosis

**Figure 4:**
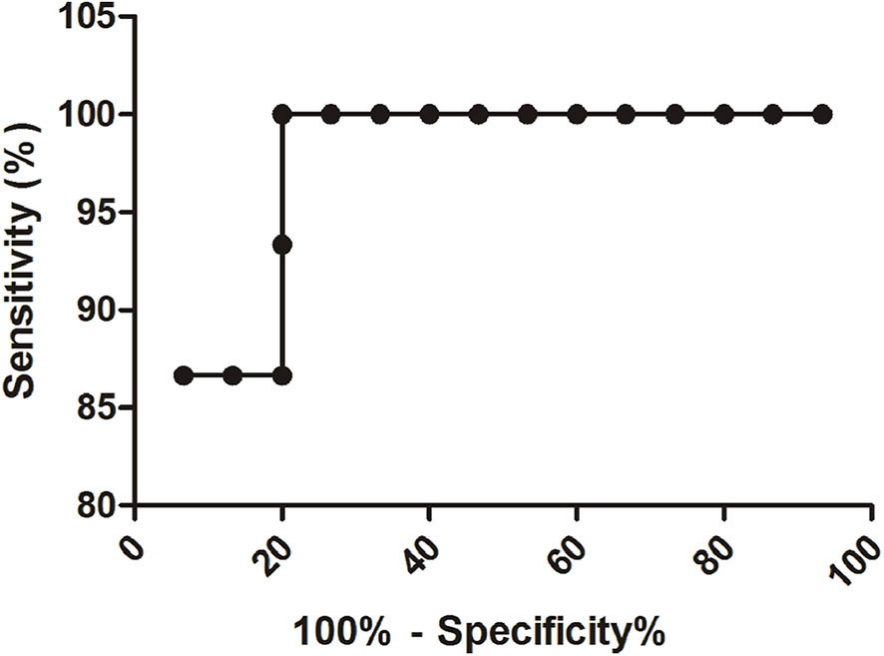
The relative importance of each input variable to the prediction for the sepsis ANN model at a clinical diagnosis.

To find the predictive power of the sepsis ANN model to discriminate between those with and without sepsis, we assessed its performance on a another part of the dataset that was not used at any time during the training, validation and testing of the model: the test cohort. The same cohort dataset was used to compare the final model performance with the clinician diagnosis (by antibiotic administration at the time of clinical suspicion). We contrasted both performances by calculating the accuracy, precision, sensitivity, specificity, predictive positive and negative values (PPV and NPV, respectively) (Table 2) from which the AUROC was obtained in the test cohort (Figure 4). Since it is preferable that control neonates are predicted to have sepsis (false positives) and not that sepsis is estimated as controls (false negatives), we also calculated the true positive rate of the model.

**Table 2:** Performance measures of the sepsis model.

**Measure**	**Physician's sepsis diagnosis (95% confidence interval)**	**ANN sepsis diagnosis (95% confidence interval)**
Accuracy	73.33% (54.11–87.72%)	86.66% (69.28–96.24%)
Precision	65.22%	82.35%
Sensitivity	100% (78.20–100.00%)	93.33% (68.05–99.83%)
Specificity	46.67% (21.27–73.41%)	80% (51.91–95.67%)
AUROC *p*-value	73.33% (54.75–91.91%) 0.0295	94.44% (85.68–100.32%) <0.0001
Standard error	0.0947	0.04373
Positive predictive value	65.22% (53.87–75.06%)	82.35% (62.70–92.83%)
Negative predictive value	100%	92.3% (63.98–98.78%)

The sensitivity of the final ANN model was 93.33% which is a very good measure of the model performance. The AUROC is also a useful tool to forecast the probability of the model, depicting the trade-off between the false positive rate (specificity) and the true positive rate (sensitivity), using different probability thresholds. The ANN sepsis model presented an AUROC of 94.4% (95% CI 85.68–100.32) in comparison with the physician's sepsis estimation value of 73.3% (95% CI 54.75–91.91) (Table 2, Figure 4).

Altogether the results from the validation and performance measures show a good correlation between the actual data and the estimation, as well as the competent predictive capacity of the sepsis ANN model.

### Importance of Maternal and Neonatal Variables for Sepsis Prediction

**Figure 5:**
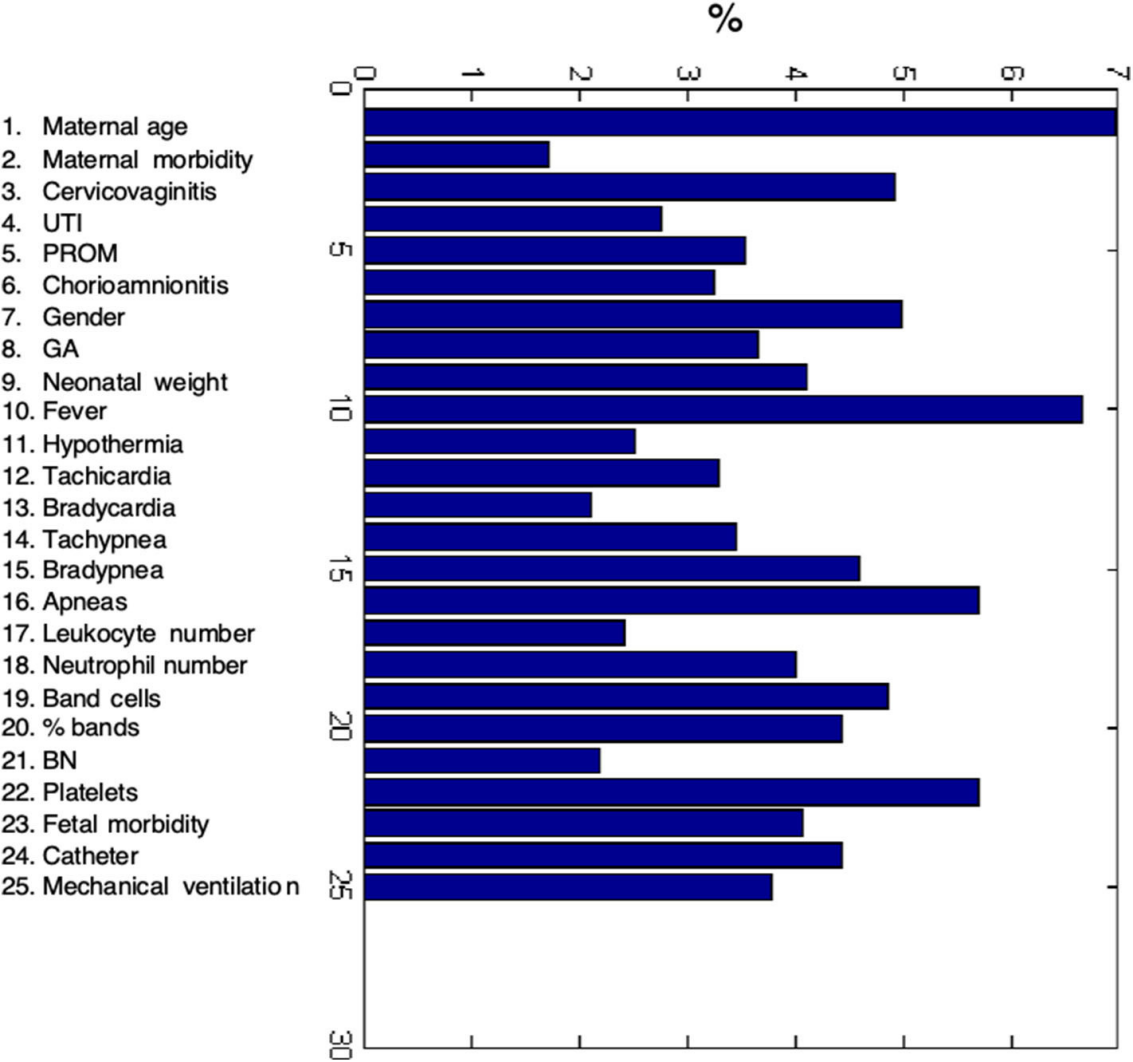
Receiver Operating Characteristics curve of the ANN model.

We used well-known features for sepsis diagnosis to train the ANN model but to determine the effect of each maternal and neonatal variable, we accomplished an evaluation process termed sensitivity analysis based on the influence of the Weights associated with each input feature. Figure 5 depicts the relative importance of each input variable (depicted as a percentage) showing that all variables had a strong effect on neonatal sepsis prediction. However, maternal age, neonatal fever, apneas, platelet counts were the most important factors for the diagnosis of sepsis followed by cervicovaginitis, gender, bradypnea, band cells (number and percentage), catheter presence, birth weight, neutrophil counts, and fetal morbidity.

## Discussion

Success in antimicrobial therapy for neonatal sepsis lays in early and accurate detection of the disease, as well as knowledge of sepsis epidemiology inside each NICU (21). However, diagnosis is still based on maternal and neonatal risk factors and unspecific clinical signs/symptoms for taking preventive actions, and unfortunately, this practice leads to an overuse of antimicrobial agents with its known consequences, such as bacterial resistance acquisition, and adverse side effects. Therefore, to reinforce clinical decision making in the NICU we developed a neural network model predicting EOS and LOS diagnosis the day of clinical suspicion based on simple maternal and neonatal factors (including known risk factors, clinical signs, and symptoms, laboratory findings), but with a particular interest in maternal and fetal/neonatal morbidities. We obtained a model with a regression coefficient of *R*^2^ = 0.974 between the actual and estimated sepsis diagnosis, and a good performance shown by a 93.3% sensitivity to correctly identify sepsis, an 80% specificity to properly find neonates without the disease, and an AUROC of 94.4% in comparison with 73.3% in physician's sepsis diagnosis (by antibiotic administration the day of clinical suspicion). This model performs optimally for a more personalized diagnosis based on maternal, fetal and neonatal morbidities in conjunction with known risk factors, clinical signs/symptoms, and laboratory findings in support of decision-making for clinicians.

Our model moderately outperforms other machine learning models for either EOS or LOS prediction with better predictive performance, however, these algorithms were designed to be forecasting while our model was developed to assist the bedside clinician, and with simple clinical criteria that identify patients with suspected sepsis the day of clinical suspicion. Nevertheless, Masino and cols. developed eight machine learning models with distinct algorithms compared to our study for EOS prediction 4 h prior of clinical recognition in preterm and term neonates (22–41 gestational age) using clinical data of sepsis suspicion and risk factors with AUROCs between 83 and 87%, NPV of 0.98, 72% specificity and PPV of 0.53 (34). A LOS prediction model within 12 h after the first blood test, performed by nine different machine learning algorithms using similar clinical variables and risk factors, obtained an AUROC, a PPV, and NPV of 92%, 0.71 and 0.76, respectively, concluding its usefulness for diagnosis support but requiring further optimization (35). A deep learning algorithm for EOS by López-Martínez group demonstrated good performance as indicated by an AUROC of 92, 80 sensitivity, 90% specificity and a PPV of 0.83 for neonates within 34–39 gestational age (23). Nevertheless, this model has some limitations with their variables, as they report a statistical difference in gestational age and weight of septic patients compared with control ones, as well as an imbalanced dataset (23). In that sense, our model was able to accurately predict both EOS and LOS sepsis diagnosis in very preterm to term neonates from a balanced dataset between septic and non-septic patients. The PPV of our model was 0.82, one of the highest reported for a prediction model, obtained from a balanced dataset.

The ANN model in this study may be compared with EOS calculators, based on gestational age and maternal risk factors such as fever, group B streptococcal presence, premature rupture of membranes and administered maternal antibiotics to predict neonatal sepsis (36–39). Unfortunately, EOS calculator does not work with neonates lower than 34 weeks of gestation. However, these calculators were designed to early predict EOS while our model was conceived to diagnose EOS and LOS the day of clinical suspicion as a tool for the clinician. Moreover, when the calculator was tested with a bigger data set, it was demonstrated that it fails to identify a significant number of EOS patients (40), however, the use of this implement has been associated with a reduction in antibiotic use (36–39).

### Important Parameters for Sepsis Diagnosis

We join worldwide efforts in proposing new early predictive tools in order to improve patient outcomes. Due to sepsis complexity, using one single biomarker in its prediction or diagnosis (except for microbial demonstration) is insufficient (20), so clinical management decisions must be taken applying a multi-disciplinary approach based on risk factors, clinical signs, and symptoms. Twenty-five maternal and neonatal factors were chosen to train the ANN model for sepsis diagnosis, including the most frequently employed in clinical practice and traditional scoring systems, well-known risk factors, anthropometric and laboratory data, but maternal and fetal/neonatal morbidities were also taken into account. Maternal factors were considered if present at third pregnancy trimester and neonatal factors at a clinical diagnosis of sepsis. The model was able to find that variables widely known as clinical features of neonatal sepsis are important for diagnosis, such as fever, bradypnea, band, platelets and neutrophil counts, in support of quality control of the algorithm (41). The top 10 parameters for sepsis prediction in our model were maternal age, fever, apneas, platelet counts, cervicovaginitis, gender, bradypnea, and band cells (number and percentage). In accordance with our results, several risk factors have been frequently studied, demonstrating their implication in sepsis development: male gender, need for artificial ventilation, gestational age <37 weeks and premature rupture of membranes (7). The strongest predictor in our model was maternal age and in agreement with other studies (35, 42) we found that adolescence is a key feature for neonatal sepsis diagnosis but we further extend this result to a maternal age >33, which predicted outcome is non-septic neonates in the presence of clinical signs and symptoms.

Cervicovaginitis, one of the described risk factors for sepsis development, was also one of the key features for the model. Such clinical manifestation is one of the main reasons for a gynecological consult during reproductive life and together with urinary tract infections (UTI), these are the most common infections during pregnancy. Even when they are reported at low percentages in the United States, its incidence reaches 30% in low-income countries (43, 44). Unfortunately, their real frequency is underestimated as 30–50% of cases are asymptomatic (45). In middle- and low-income countries including Latin America the rate varies, with some studies having found > 75% of studied women with a vaginal infection during their lifecycle (including bacteria, fungi and parasites such as Trichomonas). Therefore, this hospital performs routine cultures and cytology studies to every woman attended, and it is a requisite before any delivery procedure since the identification of asymptomatic infected women poses a great challenge during pregnancy. As cervicovaginitis, UTI have become a public health problem and had increased its prevalence during the last years; in that sense, this last feature was not found of relevance for the diagnostic tool, and this must be discussed using each country epidemiology. The least important factors were maternal morbidity, bradycardia, BN and leukocyte numbers. It is relevant to mention that since the model is predicting early- and late-onset sepsis, the relative importance of the features corresponds to learning approaches from both types of sepsis prediction.

### Limitations and Strengths of the Model

We have to acknowledge this work has limitations. The model was trained with a small but balanced database (238 neonates) from a unique center study. In that sense, we are planning to further validate the model with larger datasets from other NICUs. In addition, we are currently incorporating information from the first 2 weeks of life into our databases since neonatal sepsis is a dynamic condition, in order to train a new model of sepsis diagnosis. However, other studies have developed machine learning models with small databases with good performances, like the work by Mani and cols. with 299 neonates (35). In our work, considering the values of NPV and PPV of 0.92 and 0.82, respectively, and that NPV is the probability that a neonate is healthy given a negative prediction by the model and the PPV the probability that a patient is diagnosed with sepsis given a positive prediction by the model, these results will lead to the administration of antibiotics in false positives. Furthermore, some variables such as bradycardia, bradypnea, tachycardia, apnea, and tachypnea were converted into binary features in order to define presence or absence of these symptoms.

In spite of these limitations, our study has several strengths such as a balanced dataset, the input features are simple routinely collected and take into account maternal and neonatal morbidities, and good performance of the model in the internal (development of the model) and external validations (independent dataset).

Positive blood culture continues to be the “gold standard” for the presence of neonatal sepsis and this test is required assuming that the neonate shows clinical signs of systemic inflammation. For this reason, we used clinical symptoms of neonates with confirmed sepsis (positive blood culture) as a tool for diagnostic validation. Negative blood culture does not exclude sepsis and when the infant shows signs of infection they are considered to have clinical sepsis, and usually are treated with antibiotics. Our model was trained with neonates that presented symptoms of suspicion of sepsis, in such a way that it can be used independently of the availability of blood culture results. It is essential to mention that the non-sepsis group was defined as not-septic, with negative culture results, and included neonates with suspicion of sepsis but finally diagnosed by clinicians as not septic. Neonates with suspicion of sepsis with negative culture and diagnosis of clinical sepsis were excluded to reduce the possibility of using false negative blood culture results.

The application of the proposed model may potentially improve decision-making for early- and late-onset neonatal sepsis diagnosis in a population from extremely premature to term neonates of NICUs, based on non-specific signs and symptoms, laboratory analysis, risk factors, and maternal/neonatal morbidities, when blood culture results are not still available or without a result.

## Data Availability Statement

The datasets generated for this study are available on request to the corresponding author.

## Ethics Statement

The studies involving human participants were reviewed and approved by Institutional Review Boards of the Instituto Nacional de Perinatología Isidro Espinosa de los Reyes, Research and Ethics Committees. Written informed consent for participation was not provided by the participants' legal guardians/next of kin because: Data from medical records were collected in which patient names and identifiers were eliminated in order to have an anonymized dataset. Informed consent was not required by the Committees.

## Author Contributions

AH-R and CI contributed conception and design of the study. MS-R, SY-M, AY-M, ML-J, and JG-M collected and organized the database. CI wrote the first draft of the manuscript. AH-R, OV-C, JG-M, VZ-C and CI wrote sections of the manuscript. All authors contributed to manuscript revision, read, and approved the submitted version.

## Conflict of Interest

The authors declare that the research was conducted in the absence of any commercial or financial relationships that could be construed as a potential conflict of interest.
