# Supplementary material for: Neonatal Sepsis Diagnosis Decision-Making Based on Artificial Neural Networks
Source: Front Pediatr. 2020 Sep 11;8:525. doi: 10.3389/fped.2020.00525 (PMC7518045; doi:10.3389/fped.2020.00525)
Supplement: Supplementary file 1 [file Table_1.docx]

Supplementary Material

# Table S1. Maternal morbidity

| **Disease** |
| --- |
| 0 None |
| 1 Gestational Diabetes |
| 2 Preeclampsia |
| 3 Gestational Diabetes and preeclampsia |
| 4 Uterine myomatosis |
| 5 Leukemia |
| 6 Diabetes mellitus type 2 |
| 7 HIV |
| 8 Gestational hypertension |
| 9 Hypertension and obesity |
| 10 Hypertension |
| 11 Autoimmune disease |
| 12 Anemia |
| 13 Twin-to-twin transfusion syndrome |
| 14 Obesity |
| 15 Preeclampsia and obesity |
| 16 Gestational thrombocytopenia |
| 17 Diabetes mellitus and obesity |
| 18 Diabetes mellitus type 2, obesity and hypertension |
| 19 Pyelonephritis |
| 20 Epilepsy |
| 21 Diabetes mellitus and hypertension |
| 22 Drug addiction |
| 23 Autoimmune disease and preeclampsia |
| 24 HIV and obesity |
| 25 Autoimmune disease and hypertension |
| 26 Hypertension, preeclampsia, and obesity |
| 27 Ulcerative colitis |

# Table S2. Fetal and neonatal morbidity

| **Disease** |
| --- |
| 0 None |
| 1 Infectious pneumonia |
| 2 Anemia |
| 3 Perianal fissure |
| 4 IUGR |
| 5 IUGR and pneumothorax |
| 6 Subependymal hemorrhage |
| 7 Hydrocephaly |
| 8 RDS |
| 9 Diaphragmatic hernia |
| 10 Encephalocele |
| 11 Pneumothorax |
| 12 SDR and infectious pneumonia |
| 13 Pulmonary atresia |
| 14 Necrotizing enterocolitis |
| 15 SDR and necrotizing enterocolitis |
| 16 SDR, UTI and infectious pneumonia |
| 17 Metabolic acidosis |
| 18 Microcephalia and RDS |
| 19 Intestinal atresia |
| 20 Mycoplasma infection |
| 21 RDS and Mycoplasma infection |
| 22 Infectious pneumonia and necrotizing enterocolitis |
| 23 Renal insufficiency |
| 24 Pulmonary hypertension |
| 25 Congenital pneumonia |
| 26 Congenital pneumonia and pulmonary hypertension |
| 27 Hepatosplenomegaly |
| 28 Hepatosplenomegaly and perforated hollow viscus |
| 29 Neuroinfection |
| 30 UTI |

# Table S3. Slope and intercept values for the statistical test of the ANN sepsis model

| a_lower_  -0.0219 | a_upper_  0.0419 |
| --- | --- |
| b_lower_  0.9194 | b_upper_  1.0177 |

# Table S4. Weights and biases for the ANN sepsis model

(6 neurons in the hidden layer, k=3, and l=1)

|  | Wi_{s,k}_ | | | | | |
| --- | --- | --- | --- | --- | --- | --- |
| Wi_{s,1}_  Wi_{s,2}_  Wi_{s,3}_  Wi_{s,4}_  Wi_{s,5}_  Wi_{s,6}_  Wi_{s,7}_  Wi_{s,8}_  Wi_{s,9}_  Wi_{s,10}_  Wi_{s,11}_  Wi_{s,12}_  Wi_{s,13}_  Wi_{s,14}_  Wi_{s,15}_  Wi_{s,16}_  Wi_{s,17}_  Wi_{s,18}_  Wi_{s,19}_  Wi_{s,20}_  Wi_{s,21}_  Wi_{s,22}_  Wi_{s,23}_  Wi_{s,24}_  Wi_{s,25}_ | 2.19  0.04  0.07  -0.2  -0.7  -3.6  -3.6  2.05  -0.04  -0.8  6.2  2.31  2.5  -1.4  -0.1  -2.2  3.6  -2.9  -3.3  0.08  -0.8  -1.9  -3.8  3.21  -1.09 | 1.28  -0.7  -3.9  4.11  -2.3  -0.2  -0.4  1.63  -0.9  -0.7  -0.5  -3.3  0.05  1.65  -0.02  2.24  1.2  -0.02  -3.4  -2.8  -0.5  -1.5  -0.7  1.67  -1.8 | 0.31  -0.9  -1.5  -0.09  2.27  1.42  -0.4  -1.9  -1.5  1.1  -0.6  -0.6  -0.1  1.7  -0.3  -0.5  -1.5  -0.9  -0.7  -0.2  1.4  -0.05  -0.5  -2.1  -0.3 | 5.75  -0.6  -2.6  -0.3  -1.4  -0.3  -0.9  -0.6  3.3  -0.8  -0.8  1.31  -1.4  -0.5  2.67  3.72  1.09  -0.3  -3.8  -1.69  -2.5  7.37  -2.3  -0.9  -2.4 | -1.0  3.05  -0.7  1.26  -0.3  -0.01  4.01  -2.3  1.5  -2.5  -1.3  -0.4  -1.2  1.07  -0.9  -.015  -0.11  1.0  -0.03  -0.18  -0.6  1.3  -0.009  -0.009  -1.4 | -3.3  -0.04  2.53  -1.4  1.89  -0.8  2.82  2.64  1.66  2.02  -0.16  -0.09  -0.2  -2.9  2.5  1.6  -1.4  -2.3  -0.2  -3.86  -0.05  -0.6  -0.5  1.68  1.5 |
|  | Wo_{1,1}_ | Wo_{1,2}_ | Wo_{1,3}_ | Wo_{1,4}_ | Wo_{1,5}_ | Wo_{1,6}_ |
| Wo_{l,s}_ | -0.15 | -5.6 | 2.44 | -0.12 | -4.0 | -0.15 |
| b1_{23,1}_ | b1_{s,1}_ | | | | | |
|  | -3.95 | -1.11 | 0.24 | -1.23 | -0.52 | -4.36 |
|  | b2_{l,s}_ |  |  |  |  |  |
| b2_{1,1}_ | 2.76 |  |  |  |  |  |

**
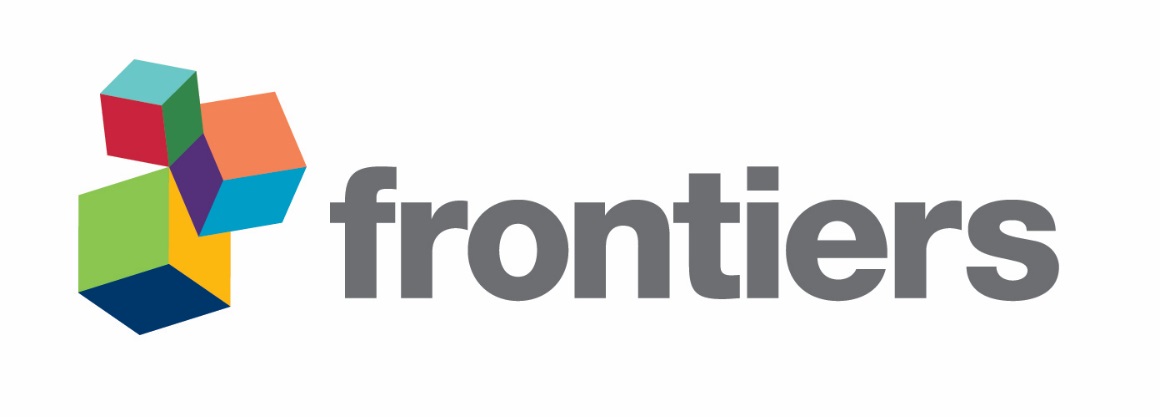
**
