# Supplementary material for: Neonatal Sepsis Diagnosis Decision-Making Based on Artificial Neural Networks
Source: Front Pediatr. 2020 Sep 11;8:525. doi: 10.3389/fped.2020.00525 (PMC7518045; doi:10.3389/fped.2020.00525)
Supplement: Supplementary file 2 [file Data_Sheet_1.docx]

Supplementary Material

**Methods**

**2.3 Training, validation and testing of the model**

The database (85% of the total after setting aside the test cohort set) was randomly split into five-equal sized parts, three parts were used for training (learning set), one part of validation (validation set), and another part for testing (test set). Training was evaluated by comparing the accuracy of the model with the validation set and this was repeated five times, each time using a different part to estimate the performance, and each part used as the test set only one time. The validation set is used to adjust the architecture of the model and to minimize overfitting by comparing the accuracy of the model in each training. Each mother/neonate is contained in only one of the splits.

For the ANN model, neurons (*n*) are associated with coefficients (Weights, *Wi* and *Wo,* and biases, b1 and b2) in the hidden and output layers, respectively (equations 1 and 2):

*N_i_* = *Wi x In*_1_ + *Wi x In*_2_ +…+ *Wi x In*_k_ + *b_1_* (1)

where *In* are the input variables. The value of each neuron is the argument of the activation functions (*f* and *g*):

*Sepsis (output) = g(Wo × f (Wi × In + b1) + b2)* (2)

Several transfer functions (hyperbolic tangent, (TANSIG), linear (PURELIN) or Log-Sigmoid (LOGSIG) were tested in the hidden and output layers.

In order to minimize the RMSE, the Levenberg-Marquardt (LM) algorithm was used with the following equation:

$\Delta w=\left( J^{T}J+\mu I \right)^{-1}J^{T}e$ (3)

Where:

*J* is the Jacobian matrix (first derivative)

*e* is a vector of network errors

*μ* is the combination coefficient (with a value of 0.001)

*I* is the identity matrix

## Evaluation of the final ANN neonatal sepsis model by performance measures: discrimination in comparison with the physician’s diagnosis

We used the following definitions:

True positive (TP, correctly culture-positive sepsis predicted), true negative (correctly control negative culture sepsis predicted), false positive (FP, culture-positive sepsis predicted but control) and false negative (FN, control culture-negative sepsis predicted but positive culture sepsis).

The following metrics were performed for the final model:

Accuracy = (TP+TN)/(TN+FP+FN+TP); (ratio of correctly sepsis predicted to the whole database) in symmetric database

Precision = TP/(FP+TP); (ratio of correct sepsis predicted to all sepsis labeled)

Sensitivity (Recall) = TP/(TP+FN); (Recall, the ratio of correct sepsis predicted to all real sepsis, true positive rate)

Specificity = (TN/(TN+FP); (correctly sepsis predicted to all control, positive predictive value)

Positive Predictive Value, PPV = TP/(TP+FP)

Negative Predictive Value, NPV = TN/(TN+FN)

**Results**

In the final model the following equation is used to diagnose neonatal sepsis:

$$Sepsis=\frac{1}{1+\exp^{-\left( n_{output\_layer} \right)}}$$

$n_{output\_layer}=\sum_{s-1}^{s} \left[ W_{o_{(l,s)}}.\left( \frac{2}{1+{exp}^{-2.(\sum_{k-1}^{k} \left( {Wi}_{(s,k)}{In}_{(k)} \right)+b1_{\left( s \right)})}}-1 \right) \right]+{b2}_{(l, 1)}$ (4)

**
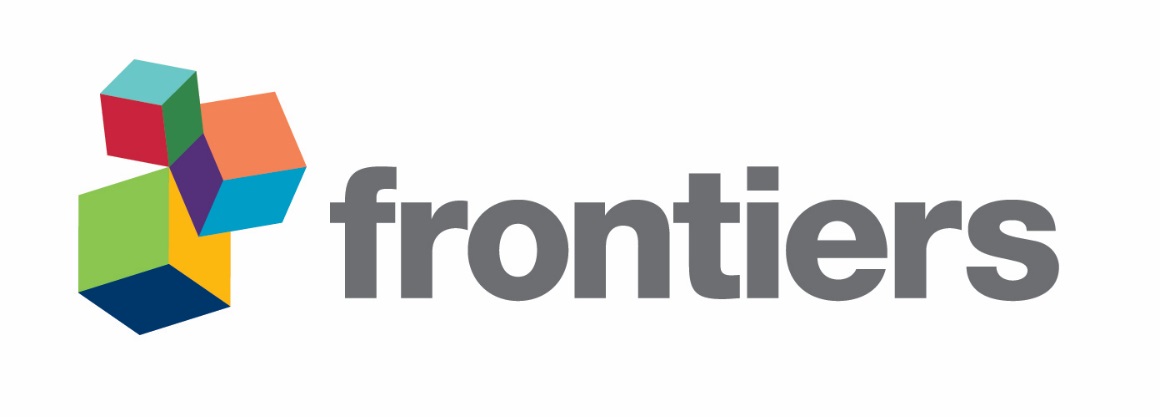
**
